# Nano-copper enhanced flexible device for simultaneous measurement of human respiratory and electro-cardiac activities

**DOI:** 10.1186/s12951-020-00632-3

**Published:** 2020-05-29

**Authors:** Li Wang, Feng Zhang, Kechao Lu, Mohammed Abdulaziz, Chao Li, Chongyu Zhang, Jun Chen, Yunlun Li

**Affiliations:** 1 Advanced Micro and Nanoinstruments Center (AMNC), School of Mechanical & Automotive Engineering, Qilu University of Technology (Shandong Academy of Sciences), Jinan, 250353 China; 2 Department of Mechanical and Process Engineering, University of Duisburg Essen, Forsthausweg, 247057 Germany; 3 Experimental Center, Shandong University of Traditional Chinese Medicine, Jinan, 250355 China; 4 The Second Affiliated Hospital, Guangzhou Medical University, Guangzhou, 510260 China

**Keywords:** Flexible device, Nano-copper, Simultaneous measurement, Respiration, Electro-cardiac signals

## Abstract

**Background:**

Dysfunction of human respiratory and electro-cardiac activities could affect the ability of the heart to pump blood and the lungs to inhale oxygen. Thus, a device could simultaneously measure electro-cardiac signal and respiratory pressure could provide vital signs for predicting early warning of cardio-pulmonary function-related chronic diseases such as cardiovascular disease, and respiratory system disease.

**Results:**

In this study, a flexible device integrated with piezo-resistive sensing element and voltage-sensing element was developed to simultaneously measure human respiration and electro-cardiac signal (including respiratory pressure, respiration frequency, and respiration rhythm; electro-cardio frequency, electro-cardio amplitude, and electro-cardio rhythm). When applied to the measurement of respiratory pressure, the piezo-resistive performance of the device was enhanced by nano-copper modification, which detection limitation of pressure can reduce to 100 Pa and the sensitivity of pressure can achieve to 0.053 ± 0.00079 kPa^−1^. In addition, the signal-to-noise ratio during bio-electrical measurement was increased to 10.7 ± 1.4, five times better than that of the non-modified device.

**Conclusion:**

This paper presents a flexible device for the simultaneous detection of human respiration and cardiac electrical activity. To avoid interference between the two signals, the layout of the electrode and the strain sensor was optimized by FEA simulation analysis. To improve the piezo-resistive sensitivity and bio-electric capturing capability of the device, a feather-shaped nano-copper was modified onto the surface of carbon fiber. The operation simplicity, compact size, and portability of the device open up new possibilities for multi-parameter monitoring.

## Background

The health data from American Heart Association in 2019 reported that more than 33% of deaths worldwide were related to heart diseases [1, 2], a great number of which are related to cardio-pulmonary dysfunction [3]. Physiological signals including electro-cardiac signals (like beating frequency, beating rhythm and electrical pulse) and respiratory signals (like respiratory pressure, respiration frequency, and respiration rhythm) have been proved that were related with cardio-pulmonary dysfunction. Accordingly, continuous and simultaneous monitoring of these signals would be beneficial in early prediction and diagnosis of these diseases. This can help patients to get timely treatment, and lower the death rate and the caring costs.

A number of devices have been conducted to record electro-cardiac signals and human respiration [4–13]. Electrocardiograph (ECG) was commonly used to describe the electrical activity of the heart. abnormal information of heart frequency and heart rhythm can be detected from ECG waveforms (e.g. P wave and QRS complex), which can be further interpreted for diagnosis of heart diseases (e.g. myocardial ischemia, myocardial infarction) [7, 14–16]. Conventional 12-lead ECG could provide comprehensive heart condition. However, the large size and the large bundle of cables make ECG only suitable for hospital-specific examinations but not for long-term and continuous monitoring of heart status [6, 17]. The early warning signals of random heart abnormality due to lacking much necessary context to the data could be missed [18]. A flexible micro/nano device is a powerful tool to solve this problem. For example, a graphene-based dry flexible electrode was used for a-week continuous recording ECG without degradation in the signal quality [13, 19].

For measurement of respiration, either pressure/strain sensing or airflow sensing methods were applied to detect respiratory info (including respiratory frequency respiratory rhythm, and respiratory pressure/volume) in recent proposed devices [6, 20–23]. However, airflow sensors need to be worn over face, which discouraged patients from long-term respiratory monitoring. The strain-depended respiratory devices were commonly attached to the surface of human chest, which could also sense other bioelectrical signals due to the co-existing physiological signals in the same region and the conductive materials used in the device (e.g. nano-gold or carbon-based nanomaterials) [24–28].

To achieve monitoring the simultaneous signals of ECG and respiration, a key technology is signal processing to separate respiratory and heat signals, using such as wavelet signal processing or principal component analysis, which have already been investigated extensively [10, 36, 37]. However, the signal processing method only provides an indicator (e.g. normalized parameter) but not a physical quantity (e.g. pressure or air inflow) for evaluating respiration, direct correlations between the indicator and the respiratory pressure produced by the lung remain to be solved. In addition, signal processing method indeed could separate respiratory signals (0.13–0.33 Hz) from ECG (1.0–1.67 Hz) and other bio-signals (e.g. haemo-dynamic fluctuations: 0.04–0.09 Hz) in human normal activity [38]. However, this method cannot distinguish the respiratory signal in abnormal state. For example, the respiratory frequency was less than 0.1 Hz when intracranial hypertension occurred [39]; another, respiratory frequency was higher than 3.0 Hz when psychopathology of hysteria occurred [40]. Furthermore, some other devices reported simultaneously measurement of respiratory signals and ECG [20]. For example, a polyvinylidene-fluoride polymer sensor patch based on the piezoelectric mechanism was developed for simultaneously monitoring heartbeat and respiration [6]. However, the devices also used an indicator to replace the physical quantity (pressure or air inflow) (Table 1), which is a similar issue with the signal processing method.

**Table 1:** The property comparisons of the current device and our device

Device	Respiration	ECG	Simultaneous measurement of respiration and ECG	Sensitivity for pressure/strain	Detection limit for pressure/strain	S/N for ECG	Refs.
Thin-film transistors	No	Yes	No	–	No	4	[18]
Wearable flexible healthcare patch	No	Yes	No	–	No	8	[29]
Flexible VDF-TrFE-CFE polymer sensor	No	Yes	No	–	No	600	[30]
CNT/PDMS composite flexible device	No	Yes	No	–	No	45.8	[31]
A stretchable carbon nanotube strain sensor	Yes	No	No	0.27 (for strain)	0.16	No	[32]
Wearable strain sensor	Yes	No	No	0.03(for pressure, kPa)	0.1443	No	[33]
Wearable physiological monitoring	Yes	Yes	No	7.2 (for strain)	0.125	3	[34]
Self-powered flexible pressure sensor	Yes	Yes	No	0.2	0.25	10	[35]
Our device	Yes	Yes	Yes	0.053	0.1	10.7	This study

In this study, we proposed a flexible device that can simultaneously measure ECG and respiration (including parameters of electro-cardio frequency, electro-cardio rhythm, and electro-cardio amplitude; respiratory frequency, respiratory stress, and respiratory rhythm). Nanoparticle modification and finite element analysis (FEA) were used to improve the performance of sensing elements and optimize the structure of devices [41–45]. The key improvement of the device is that the carbon fiber was modified with nano-copper. The modification of nano-copper onto electrochemical methods has excellent performance in improving the sensitivity and the detection limit of the analyte [46–48]. However, the piezo-resistive sensitivity and of electrical capturing ability of the nano-copper have not been reported. Finite element analysis (FEA) was used to optimize the structure of device. For the devices with monitoring functions of respiration and ECG, FEA optimized the layout of each sensing element from the following considerations [49]: (1) The microelectrode for measuring ECG should locate at the place with small strain, which can avoid the movement of microelectrode during breath [50]. (2) In addition, the distance between the electrode and the piezoresistive strain sensor should be determined. This can reduce the effect of the transient electrical field on detecting resistance signals during the electrical pulse generated by the heart [51]. To obtain stabilized output, the device was preconditioned for 4500 s. The relationship between the resistance change and the pressure was also calibrated, which would be used for converting the resistance change in the measurement to the pressure of human respiration. Finally, 17 volunteers were tested to prove the suitability of the device for simultaneously monitoring of ECG and respiration.

## Results and discussions

### CNT-PDMS device characterization

#### FEA result of the flexible device

The simulation of the stress distribution and the simulation of the electric field distribution were used to guide the layout of the flexible device.

**Fig. 1:**
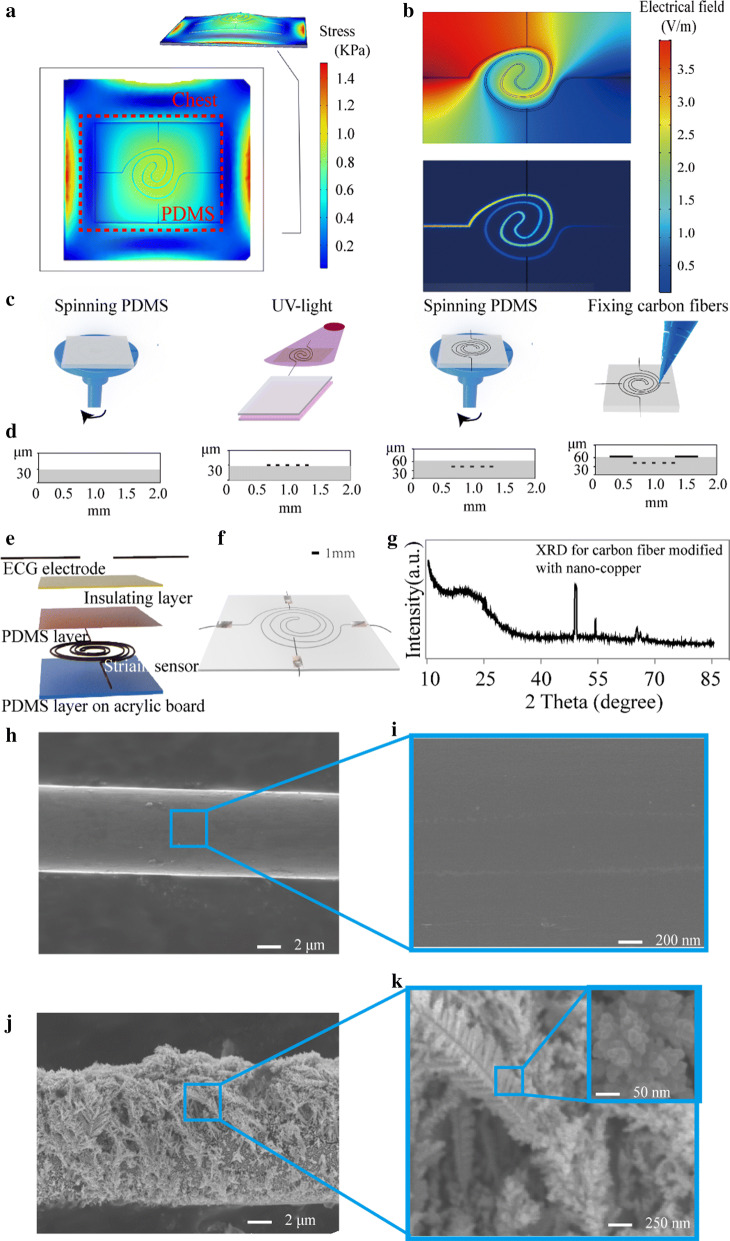
Simulations for guiding the layout of the flexible device and characterization of the fabricated device. **a** Stress simulation of and strain sensor in the flexible device. The stress gradually decreased from the center of the device along the radial direction, and stress concentration occurred at the fixed edges of the device. **b** The electrical field simulation of the electrode in the device with and without insulating layer. The up-top inset revealed that the electrical field could transport across the whole device. The bottom inset showed that electrical field only exists along the strain sensor. The electric field strength of other locations, especially around the microelectrodes were nearly 0 V/m after adding an insulating layer. **b** The fabrication process of flexible device. First a thin PDMS film was spin-coated onto a 2 cm × 2 cm acrylic slide. Then lithography was employed to form a spiral channel and carbon fiber was assembled into the channel. The isolation layer was spin-coated onto the carbon fiber. Finally, carbon fiber adhered to the top layer of PDMS. **d** Determination of flexible device dimension during its fabrication. **e** The schematic for the developed device. **f** An image of the fabricated flexible device. **g** XRD for determining the existing of carbon fiber and nano-copper. **h** SEM of bare carbon fiber. **i** SEM of enlarged bare carbon fiber for showing its smooth surface. **j** SEM of and the carbon fiber modified with nano-copper (electroplating time = 80 s). **k** SEM of enlarged carbon fiber modified with nano-copper, which showing nano-copper with feather-shape. The inset showed that the feather-shaped nano-copper was constructed by spherical nanoparticles

For mechanical stress simulation, a pressure (1.5 kPa) was applied onto the whole device. The edges of the device were set as fixed, and the other parts of the device were set as free. The young’s modulus of the floating PDMS membrane was 467.5 kPa ± 10.27 kPa measured by AFM. The simulation result (Fig. 1a) showed that the stress gradually decreased from the center of the device along the radial direction, and stress concentration occurred at the fixed edges of the device. The corresponding stress values decreasing from 0.51 kPa to 0.06 kPa. The results also revealed that the layout of the electrodes for ECG should avoid the locations of the stress concentration and the center of the device. This arrangement could reduce the influence in detecting ECG caused by the shape change of the device during the movement.

As resistance change was performed for detecting human respiration, a continuous voltage (0.5 V) was applied to the strain sensor. The electrical field generated by the applied voltage may interfere with cardiac electrical pulse detection. Based on the consideration, an insulating layer was added between the microelectrode and the strain sensor. To prove the efficiency of the insulating layer, the electrical field of the device with and without insulating layer was simulated (Fig. 1b). The top inset in Fig. 1b is the electrical field distribution of the device without insulating layer. The result showed that (1) the electrical field could transport across the whole device, thus it could interfere with the detection of the cardiac electrical pulse. (2) The electrical field covered all the top layers of PDMS, and electrical field unevenly decreased from the positive pole (4.5 V/m) to the grounding pole (0 V/m). However, the bottom inset in Fig. 1b showed that electrical field only exists along the strain sensor. The electric field strength of other locations, especially around the microelectrodes were nearly 0 V/m after adding an insulating layer.

#### Device characterization

To characterize the geometry of the device, an optical profiler (Bruker, USA) was employed to scan the top-surface morphology during steps of fabrication. The thin PDMS film was the critical component in the device, which thickness determines the performance in sensing the strain caused by respiration. To make the first layer of PDMS film with a thickness of 30 μm, the PDMS was spin-coated onto photoresist (AZ4620) at the speed of 1600 r/min as shown in the left-first plot of the Fig. 1d. After locating carbon fiber into photoresist, the thickness of the device increased to 37 μm, as shown in the left-second plot of Fig. 1d. Then, the thickness of the second PDMS layer was spin-coated at the above same speed, which was around 30 μm. The total thickness of the device was around 67 μm after locating ECG electrode.

XRD was used to determine the existence of nano-copper and carbon fiber, which is the direct evidence for the periodic atomic structure of a specific element [52]. An inner-section of a flexible device was identified by XRD with scan the 2θ degree from 10° to 85° (Fig. 1g). The spike at the 2θ degrees of 42.5°, 51°, 45°, and 73° represent the existence of nano-copper. Similarly, the spike at the 2θ degree of 22 was the characteristic peaks of carbon fiber. The results demonstrate that the nano-copper was modified on the surface of carbon fiber.

Then, the 2D surface morphology of carbon fibers with and without nano-copper was captured by SEM (Fig. 1h–k). Figure 1h shows that it displays like a cylindrical stick with a diameter of 7.1 μm ± 0.2 μm. Figure 1i shows that the tidy and smooth surface of carbon fiber, which is a benefit for nano-copper to adhere. For the carbon fiber modified with nano-copper (Fig. 1j), the diameter increased to 8.0 μm ± 0.2 μm, and the enlarged SEM (Fig. 1k) shows that numbers of feather-shaped nano-copper are grown on the surface of carbon fiber. The inset in Fig. 1K shows that the feather-shape nano-copper consists of numbers of spherical nanoparticles, which diameter concentrates at 100 nm. These nanoparticles can greatly improve the specific surface area of the sensor, which can improve the sensitivity of the sensor and lower detection limit [53].

### Mechanical behaviors of the flexible device

#### Improvement of mechanical response after flexible device modified with nano-copper

**Fig. 2:**
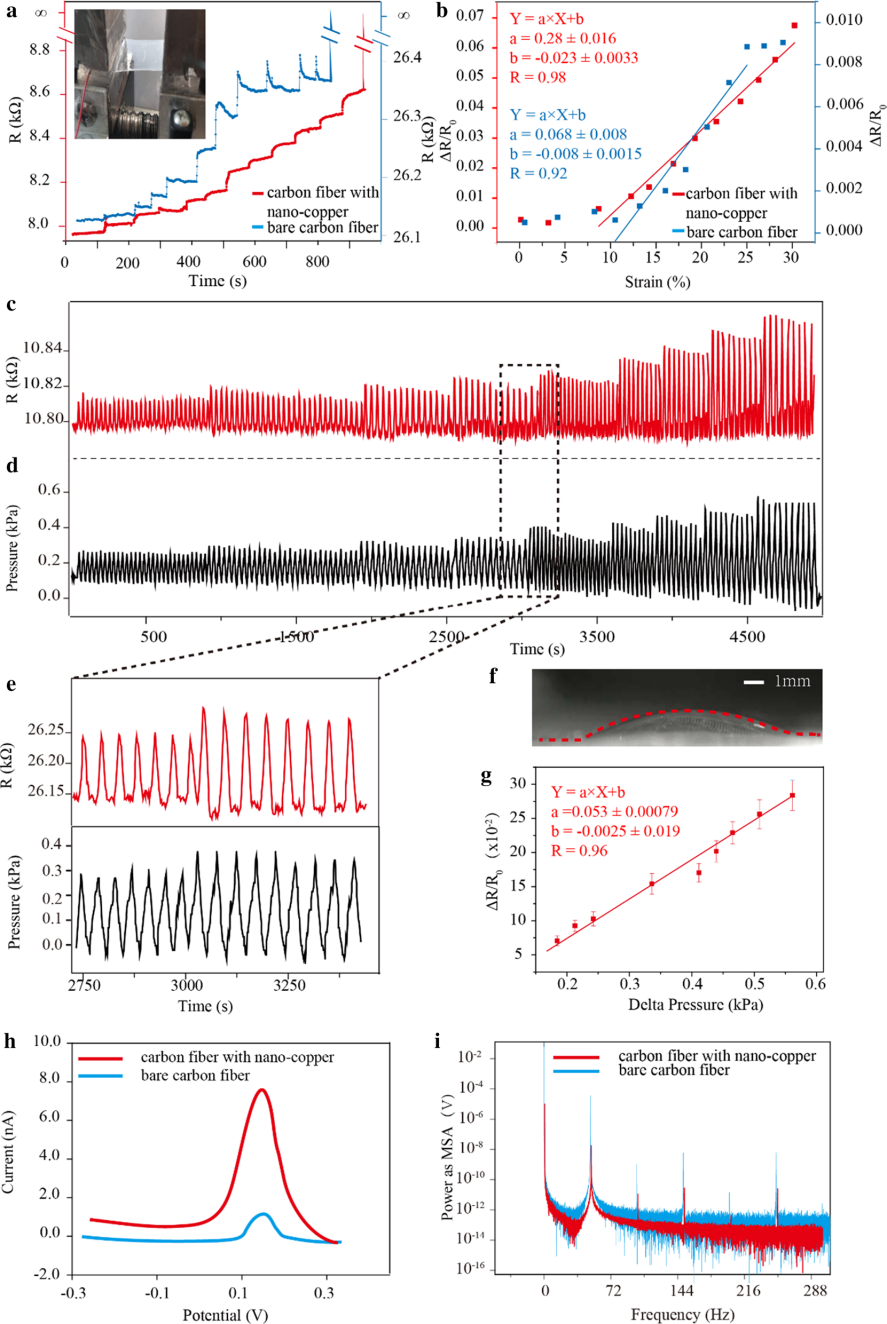
Mechanical and electrical properties of the flexible device. **a** Tensile failure testing of carbon fiber with nano-copper and bare carbon fiber. The strain changed from 2.5 to 32.5%. The carbon fibers were all broken up at a strain of 32.5%. **b** The scattered points between ΔR/R_0_ and strain. The strain sensitivity of carbon fiber with nano-copper was 11.82 times than that of bare carbon fiber. **c**, **d** The dynamic resistance change of the device under the applied dynamic pressures. **e** The enlarged plot for showing resistance change when pressure changed from 0.35 to 0.40 kPa. **f** The bulged flexible device under pressure of 0.6 kPa. **g** The fitted curve between and pressure for calculating sensitivity and linear ship (sample number = 4). **h** Differential pulse voltammetry (DPV) for evaluating the stored charge ability of carbon fiber with nano-copper. **i** Fourier transform for evaluating the anti-noise ability of carbon fiber with nano-copper

To compare the mechanical behaviors between the flexible device with nano-copper and the device without nano-copper, the strain testing was first performed. Figure 2a shows the resistances of the devices with and without nano-copper changed with a gradual increase of strain. The resistances of both devices increase with each 2.5% increment of the strain. Under the same strain, the resistance response (ΔR/R_0_) of the flexible device with nano-copper was around 12-fold than that of the flexible device without nano-copper. For example, the resistance response (ΔR/R_0_) of the flexible device without nano-copper was 0.0011 under the strain of 10%, whereas ΔR/R_0_ of the flexible device with nano-copper was 0.013. Furthermore, the strain-resistance curve was fitted, Fig. 2b shows the linear range of the modified flexible device is from 7.5 to 30%. However, the linear range of the flexible device without nano-copper was from 10 to 22%. The results revealed that the mechanical response (strain sensitivity and linear range) of the device could improve through modifying nano-copper.

#### Tensile failure and response time of the device with and without nano-copper

Both devices were broken when the strain increased to around 32.5%, this result was similar to the breaking point of PDMS without carbon fiber [54]. This indicates that PDMS embedded with individual carbon fiber (diameter = 7 μm) would not affect the tensile performance of flexible devices.

In addition, we found that the flexible device with nano-copper would prolong the response time to stabilize resistance for each strain change. For example, when the strain of the thin PDMS membrane is greater than 10%, the stable time of flexible device with nano-copper needs more than 20 s. However, the response time of a flexible device without nano-copper needs no more than 10 s. This result may be attributed that many nano-copper nanoparticles overlapped on the surface of carbon fiber (verified from the SEM in Fig. 1k) of nano-copper, which is not stable when the device undergoes strain. To overcome the issue, preconditioning for each fresh fabricated device would be performed as follows.

#### Preconditioning strain sensor

To stabilize the electrical resistance of the modified flexible device within the measurement period (human respiration period is around 4–6 s), each fresh device was firstly preconditioned for 4500 s under the strain of 7.5%. Additional file 1: Fig. S1A showed that electrical resistance of flexible devices with nano-copper changed over time similarly in logarithmic growth way. The electrical resistance increased from 7.96 to 10.82 kΩ. With preconditioning going on, the electrical resistance of the carbon fiber with nano-copper has a little change (from 10.82 to 10.84 kΩ), and electrical resistance (ΔR/R_0_) has no obvious change (from 0.00924 to 0.00923), which indicates that the response of device with nano-copper would be stable after preconditioning 4500 s.

### Calibrating relationship between respiratory pressure and ΔR/R_0_

For quantifying respiratory stress using the developed device, the relationship between resistance change (ΔR/R_0_) and corresponding stress caused by respiration was necessary to be established [55]. To this end, a device was assembled onto the surface of an open cylinder (Additional file 1: Fig.S1B), thin-film in the device would bulge/concave when the air was imported/exported with a micro-pump. The electrical resistance of carbon fiber would change due to the shape change (strain change) of the thin film [56]. Figure 2c showed that their electrical resistance stepped increased when pressure changed from 0.1 to 0.6 kPa. When the applied pressure (the delta pressure of 0), the resistance change (ΔR/R_0_) under was 0. The signal-to-noise ratio between resistance and background noise was larger than three when 100 Pa was applied, which was regarded as the detection limit of the strain sensor (the corresponding ΔR/R_0_ was 0.0034). Figure 2e was the enlarged plots of resistance response and the input pressure when the pressure changed from 0.35 kPa to 0.40 kPa (the delta pressure of 0.05 kPa). For obtaining a steady piezo-resistive response, each pressure was recycled 30 times. The data was extracted and fitted as shown in Fig. 2g. The calculated sensitivity of the device was 0.053 ± 0.00079 kPa^−1^ with a fitting coefficient of 0.96.

### Electrical performance of the modified carbon fiber

#### Stored charge capacity of the electrode in flexible device

The ability of store charge of the microelectrodes in the device plays an important role in sensing the weak ECG [16]. To test this ability, electrochemical method (Differential pulse voltammetry, DPV) was used. The microelectrodes were scanned from − 0.3 to 0.3 V at the speed of 50 mV/s in the 0.05 M solution of potassium ferricyanide (K_3_Fe(CN)_6_), as shown in Fig. 2f. The area of the CV curve represents the charging-discharging performance. The area of microelectrode with nano-copper was 6.6 times larger than that of microelectrode without nano-copper (8.0 nA vs. 1.2 nA). This demonstrates that the ability of store charge would largely increase by modification of nano-copper. This is due to that many porous nanoparticles increase specific surface area and increase electron transfer speed [57].

#### Anti-background noise ability

Electrical background noise is another factor that would affect the detection of ECG [58]. The reported amplitude of ECG often ranges from 0.1 to 10 mV. If the introduced background noise was larger than this value, the device will not observe the effective ECG signal [59]. To this end, we performed anti-noise testing. Additional file 1: Fig. S2E showed the time domain graph of the introduced background noise. The amplitude of the microelectrode without nano-copper was 3.45 ± 0.68 mV, however, the amplitude of the microelectrode with nano-copper was 0.37 ± 0.09 mV. In the meanwhile, 50 Hz power line was the primary interference from spectral analysis (Fig. 2i).This is because that the nano-copper carries with numbers of charged active-particles on the surface, which can increase the electron transfer speed and improve the anti-interference performance [60].

### Optimization of modification time and exploring mechanisms for sensing strain and voltage

#### Optimization and strain-sensing mechanism of the strain sensor

**Fig. 3:**
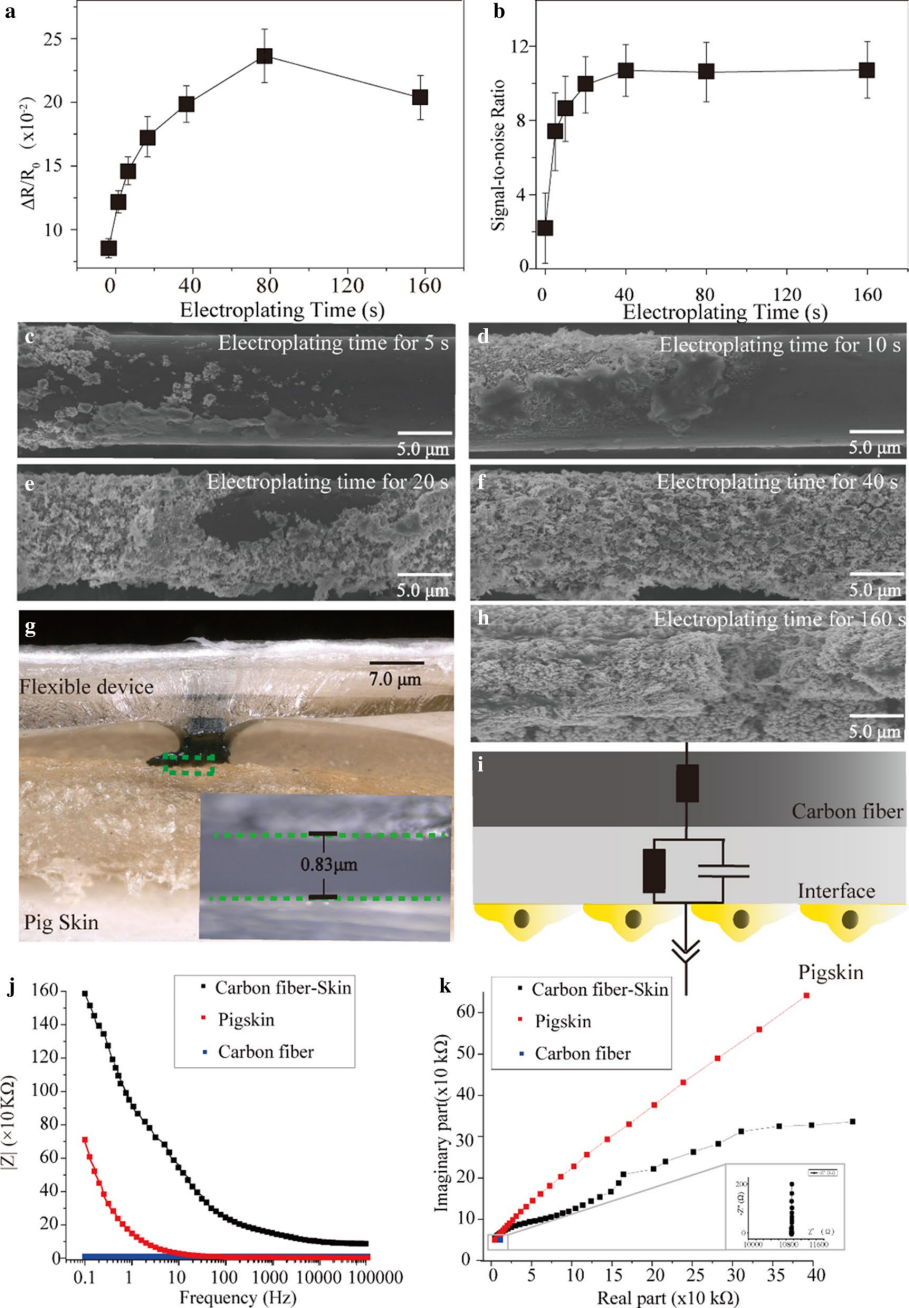
Optimiztion of the nano-copper modification condition and exploration of voltage-sensing mechanism the nano-copper **a**, **b** optimization for modification condition of carbon fiber. Results showed the 80 s and 40 s were respectively optimized conditions for modifying strain sensors and microelectrode. **c**–**f**, **h** The SEM images of carbon fiber modified under different electroplating times. It contributes to explain the piezo-resistive response for the carbon fiber. **g** The model for testing the frequency response of carbon fiber and pigskin. **i** An electrical model for decoupling voltage-sensing mechanism. **j**, **k** Bode plots and Nyquist plots for decoupling the specific values in the electrical model

To enable the strain sensor and the ECG electrode of the device with the optimized performance, the modification time of the nano-copper was explored. Six groups of modification time (5 s, 10 s, 20 s, 40 s, 80 s, and 160 s) were used. Resistance changes (ΔR/R_0_) of the modified devices were measured under the pressure change of 0.51 kPa. Figure 3a shows that ΔR/R_0_ achieved to the largest value (0.023) at the modification time of 80 s, then ΔR/R_0_ decreased with the increase of modification time. The resistance change of carbon fiber with nano-copper is determined by the distance between adjacent nano-copper nanoparticles and the conductive path formed by the inter-connections of nano-copper. When an external force or pressure is applied on the nano-copper, the distance between the adjacent nano-copper becomes larger, the formed nano-copper conductive path can be broken, which was similar to the composites of CNT and PDMS [61–63]. Accordingly, the chance of electronic transition between adjacent nano-copper is reduced. The morphologies of nano-copper under the different electroplating time were captured by SEM to explore the strain-sensing mechanism (Fig. 3c-f, h). Only a few nano-copper was modified on the surface of carbon fiber when electroplating time was 5 s. With the increase of electroplating time, the surface of nano-copper was gradually covered. Until the electroplating time was 80 s, a uniform layer of nano-copper was grown on the surface of carbon fiber. However, the blocky-shaped particles appeared and irregular surface lead to an increase of roughness when the electroplating time increased to 160 s.

#### Optimization and voltage-sensing mechanism of the ECG electrode

To obtain the voltage signal with anti-noise ability, the modification time for the ECG electrode was also explored. Signal-to-noise-ratio (S/N) was used to evaluate the performance of each electrode under different conditions. The curve in Fig. 3b showed that S/N increased with the increase of electroplating time. When modification time was 40 s, the S/N trends to be steady with the value of 10.7 ± 1.4, which has no significant difference with the S/N observed under modification time of 80 s and 160 s. Thus, 40 s were selected as the modified condition for micro-electrode.

To explore the mechanism of sensing ECG, an equivalent circuit model between the microelectrode and the surface of the skin was proposed. As the physiology of pigskin resembles human skin [64], it was used to simulate human skin (a flexible device was located onto the surface of pigskin, Fig. 3g). The frequency responses (Bode plots and Nyquist plots) of pigskin, microelectrode, and the integrity of the pigskin and microelectrode were respectively measured from 0.01 to 100 kHz (at 20 mV), which were shown in Fig. 3j, k. Bode plot of carbon fiber (blue curve in Fig. 3j) showed that carbon fiber is a resistance with the value of 10.87 kΩ that will not change with frequency change. The Nyquist plot (red curve in Fig. 3j, k) shows that frequency response of pigskin is a straight line with slope of 0.45, which demonstrates that pigskin behaves as a constant phase element. The Nyquist plot of the black curve in Fig. 3k shows that a semi-circle appears when the carbon fiber electrode attaches to the surface of pigskin, which corresponds to the gap between flexible device and pig skin (the inset in Fig. 3g). It can be regarded as the parallel of the resistance and capacitance in the circuit model, and the value of capacitance is 1.6 μF ± 0.41 μF (Fig. 3i).

### Simultaneous measurement of respiration and electrical activity from the human body

**Fig. 4:**
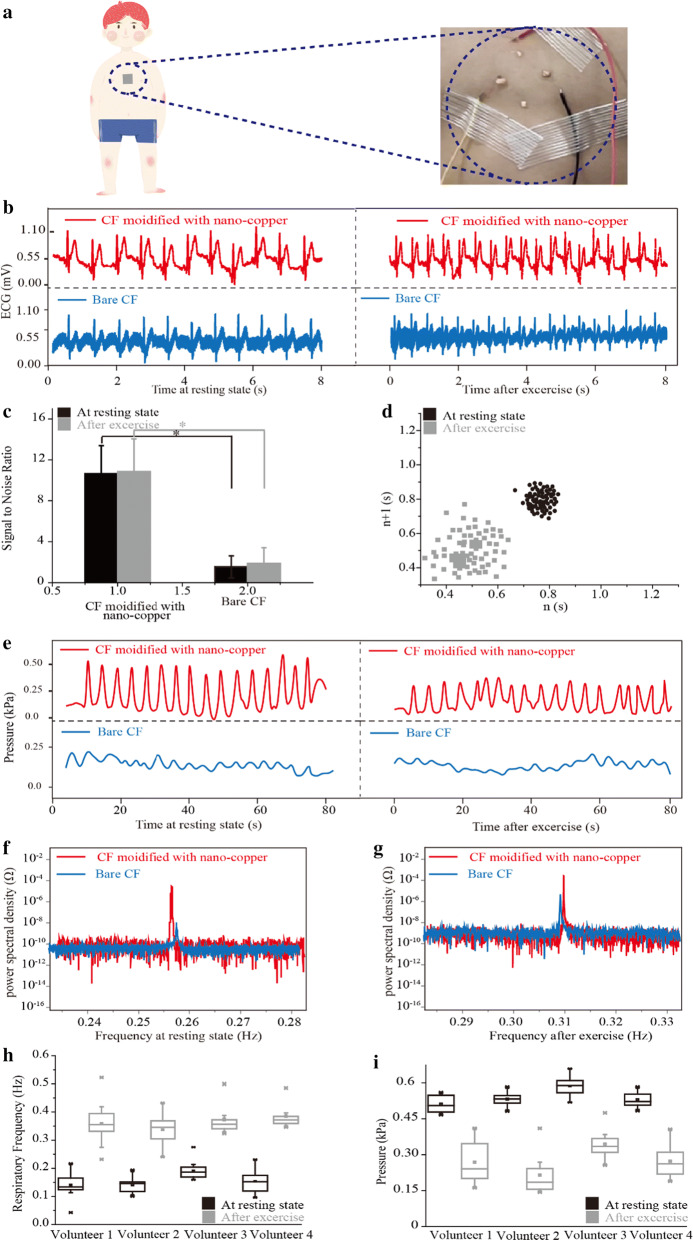
Simultaneous measurement of respiration and ECG of the volunteers at resting state and after exercise. **a** A demonstration of a flexible device adhered to the surface of chest skin. The enlarged figure for showing our device with good adhesion and transparency. **b** ECG obtained by carbon fiber (CF for short) with nano-copper and bare carbon fiber. The ECG signals were also obtained before and after jumping 50 times. **c** Statistics of signal-to-noise ratio for comparing the quality of the signals obtained by carbon fiber with nano-copper and bare carbon fiber. Results showed there is a significant difference in signals obtained by carbon fiber with-nano-copper and bare carbon fiber. *P-value < 0.05. **d** Point-care plot for showing the beating period change before and after exercise. **e** Respiratory signals obtained by carbon fiber with nano-copper and bare carbon fiber. The signals were also observed before and after exercise. **f**, **g** Fourier transform for showing the energy distribution before and after exercise. The blue curve represents the distribution of bare carbon fiber, the red curve represents the distribution of carbon fiber with nano-copper. **h**, **i** Box charts for showing the change in respiratory frequency and respiratory stress before and after exercise

To further verify the ability of our device that can measure respiration and electrical activity, 17 volunteers were employed to simultaneously record breath and heart electrical activity before and after exercise. The volunteers’ age ranged from 18 to 31, with a height from 55 to 75 kg. The video of Additional file 2 and Fig. 4a showed that a volunteer was monitored by our developed flexible device, the rhythmical ECG and resistance signals can be simultaneously observed at resting state. The top-left plot in Fig. 4b shows that the ECG signal sample was recorded from a volunteer at the resting state by a carbon fiber modified with nano-copper. The top-right plot in Fig. 4b shows that the same carbon fiber was recorded ECG from the volunteer after jumping 50 times. We also used the carbon fiber without nano-copper to measure ECG signal from the volunteer (see the bottom-plots in Fig. 4b). To quantify the difference of ECG signals observed from two kinds of carbon fiber, statistics for the signal-to-ratio (S/N) was proceeded (Fig. 4c). At resting state, the S/N of the microelectrode with nano-copper was 10.7 ± 1.4, and the S/N of the bare microelectrode was 2.2 ± 1.9. The *P* value was 0.007, demonstrating that significant difference exists between the two kinds of carbon fiber. After exercise, both S/N of the carbon fiber with and without nano-copper increased a little (carbon fiber with nano-copper increased 4.86%). To clearly characterize the heart state change before and after exercise, Poincaré plot was introduced. Poincaré plot was described by neighboring beating periods, thus it can reflect whether the heart has rhythmic beating. From Fig. 4d, we can see that the beating period gather together about 0.63 s at the resting state, after exercise, the beating period shortened to 0.45 s and arrhythmic beating occurred in this volunteer.

In addition, respiratory signals were also analyzed. The parameters including respiratory frequency, respiratory pattern, and respiratory stress were quantitatively analyzed [65]. At resting state, the respiratory frequency was 0.13 Hz, respiration pattern displays as an asymmetrical triangular wave. Respiratory stress changed from 0.46 to 0.57 kPa. As a comparison, carbon fiber without nano-copper was also employed to measure respiration [23]. This kind of carbon fiber could capture the respiration at the resting state and after exercise. However, the amplitude was much less than that observed from the carbon fiber with nano-copper (0.21 kPa vs. 0.57 kPa). Similarly, S/N was also less than that of the electrode with nano-copper (7.3 vs. 10.2). We used Fourier transform to compare the energy distribution with and without nano-copper (Fig. 4f). At resting state, the power magnitude of the respiratory signal measured by nano-copper modified electrode was 5.20 × 10^−5^ Ω, whereas the magnitude of the respiratory signal measured by bare electrode was 1.01 × 10^−8^ Ω. After exercise, the magnitude of respiratory signal measured by nano-copper modified electrode was 3.18 x 10^−4^ Ω, the magnitude of respiratory signal measured by bare electrode was 7.44 x 10^−6^ Ω. Furthermore, we analyzed four of 17 volunteer’s respiratory frequency and respiratory stress using box plot (Fig. 4h, i). For respiratory frequency, the four volunteers have obvious difference before and after exercise, with the range changing from 0.12 ± 0.11 to 0.38 ± 0.14 Hz. Respiratory stress represents the strain change of flexible device caused by the inspiratory volume of air. Thus, we compared the four volunteer’s respiratory stress before and after exercise. All amplitude of respiratory stress decreased after exercise, which specific values of respiratory stress changed from 0.55 ± 0.09 to 0.28 ± 0.12 kPa.

## Methods

### Simulation of strain distribution and electric field intensity

Finite element analysis (FEA) was employed to optimize the structure of a flexible device [66]. The simulation result would contribute to optimizing the layout of sensing elements (microelectrode and strain sensor) in flexible devices. The elastic modulus of the flexible device, dimensions of the device and applied pressure were used as input data for the FEA model. The elastic modulus of the flexible device was 467.5 ± 10.27 kPa (n = 6), which was measured by Atomic Force Microscopy (AFM, Bruker, America) (Additional file 1: Fig.S3A). PDMS was often regarded as the isotropic elastic materials in flexible device, and which Poisson’s ratios was 0.49 in simulation model [67]. Optical profiler (Bruker, USA). The applied pressure was 1.5 kPa, which was measured from a volunteer using a pressure meter.

To determine whether the applied voltage in the strain sensor affects the detection of heart electrical signals, the distribution of electric field was simulated. A 2-Dimensional axisymmetric geometry was used as the simplified model. The material of flexible device is selected as PDMS, and its conductivity was defined as 10^−3^ S/m. The conductivity of the electrode with nano-copper is defined as 10^6^ S/m, which relative dielectric constant is 12. The remaining parameters are processed by the default values of the material properties in the software [68].

### Fabrication of the flexible device

The developed device could simultaneously sense electrical pulse and strain change, which is achieved by the microelectrode and piezo-resistive element [66]. Figure 1c (The detailed fabrication process in Additional file 1: Fig. S2) shows the fabrication process: Acrylic slide with thickness 2 mm was selected as a substrate, and it was washed following acetone, ethyl alcohol, and deionized water. Then, we calibrated the relationship between the film’s thickness and spin-coating speed. As the viscosities of PDMS and photoresist (AZ4620) are different, Additional file 1: Fig. S3C, D showed their thickness decreased with exponential decay. The results are similar to the Ref. [69]. Through the above relationships, positive photoresist (AZ4620) was spin-coated for 1 min at the speed of 1000 r/min (Before spin-coating AZ4620, the PDMS thin film should be cleaned by plasma). After solidification, A mask with a spiral pattern was placed onto the AZ4620 and was exposed to UV light (wavelength: 365 nm) for 23 s using a lithography machine. After that, the spiral channel was patterned in the layer of photoresist, the carbon fibers (the nano-copper modified or the unmodified) were gently embedded into the channels. Polydimethylsiloxane (PDMS, Sylgard 184, Dow Corning, US) with a weight ratio of 1:10 (curing agent to prepolymer) was spin-coated for 1 min with speed of 1600 r/min, and it was baked in the oven at 65° for 40 min [70]. This could form a thin PDMS film with thickness of around 30 μm. Then, the PDMS doping with carbon nanotube was spin-coated to be insulating layer. The second PDMS was pin-coated onto the insulating layer at the 4000 r/min. The total thickness of the insulating layer, and the second PDMS film was about 30 μm. When the second PDMS film was half-solidified, the carbon fibers modified with copper nanoparticles (ECG electrode) were assembled to the surface of PDMS layer. At each end of carbon fiber, the copper wire was weld by an electric iron. After that, the curing process was continued by baking for 4 h. Finally, the device with PDMS was immersed in the acetone for dissolving positive photoresist (AZ4620), a flexible device with microelectrodes and strain sensors was fabricated as shown in Fig. 1f.

### Electrochemical synthesized copper nanoparticles

The electrochemical modification of copper nanoparticle was performed as follows [56]. In a typical procedure, carbon fiber was firstly washed by oxygen plasma for 15 s at the power of 50 W. Then, 0.5 g copper sulfate (CuSO_4_) was dissolved in 100 mL 0.1 M solution of sodium nitrate (NaNO_3_). Carbon fiber, platinum, and Ag/AgCl were respectively used for a working electrode, a counter electrode, and a reference electrode. The electrodeposition was carried out by chronoamperometry for 50 s at the voltage of 12 V at room temperature. To obtain the uniform copper nanoparticle on the surface of carbon fiber, the carbon fiber was slowly inserted into the reaction chamber with a fixed speed of 5 mm/s [71]. After that, the modified carbon fiber was assembled into the PDMS.

### Characterization of the flexible device

Optical profiler (Bruker, USA), SEM (Hitachi, Japan), AFM (Bruker, USA), and XRD (Rigaku, Japan) were employed to characterize flexible devices. The optical profiler was used to obtain geometric dimensions of the device during fabrication. In each step of fabrication, the thickness and width of the device were investigated, and which would contribute to constructing a geometry model used in the simulation. Before fabricating device, the thickness of the PDMS was also measured by optical profiler for correlating with spin-coating speed [72]. Atomic force microscope (AFM) and SEM were used for observing 2D and 3D surface morphologies of individual particles and groups of particles. AFM offers the visualization in diameters of nanoparticles. X-ray Powder Diffraction (XRD) (Rigaku, model Geigerflex) was used for phase identification of PDMS, carbon fiber and nano-copper and can provide information for determining the existence of the three materials. The diffractograms were measured at a scanning speed of 8°/min, by means of a tube voltage of 40 kV and a tube current of 30 mA [73].

### Tensile failure test and preconditioning of flexible device

Tensile failure is the maximum tensile stress that a device can take. The test was performed to the flexible device embedded with nano-copper modified carbon fiber and the device with unmodified- carbon fiber. First, both ends of the flexible device were fixed into two PDMS blocks (the inset in Fig. 2a). Then, a home-made stretching instrument (Additional file 1: Fig. S3B) closely clamps PDMS blocks and stretches them gradually, as shown in the video in Additional files 3—the preconditioning process of the proposed flexible device by a home-made stretching instrument. In each step, the stretching instrument increases 2.5% strain through programmable procedure, and keep it at least 120 s for obtaining the stable resistance. The changes in strain and resistance were recorded until the flexible device broke down. To obtain the stable resistance-response for strain change, we performed preconditioning (tensile training) for each device (including the carbon fiber modified with nano-copper and the bare carbon fiber). Through analyzing the curves between strain and resistance, the first point in the linear range of the curve was selected for tensile training. The resistance was continuously recorded until the stable response of the strain sensor was observed. The sampling rate was 100 Hz. For each device, the tensile training was performed along portrait orientation and landscape orientation.

### Calibration of the flexible device

To predict the pressure generated by respiration, the relationship between pressure and resistance should be calibrated. We sealed a device onto the surface of a cavity and inserted a needle into the cavity from side face. A homemade microinjection pump was used to bulge the flexible device, and a Y-type tee was connected with pressure meter (accuracy = 1 Pa, Hong Kong) (A schematic was shown in Additional file 1: Fig. S1B). The resistance change and pressure were simultaneously recorded during flexible device were bulged by the pump. The input pressure changed from 100 to 600 Pa and kept for 2 min at each pressure. The sampling rate was 100 Hz. The bulged shape of flexible device was also captured by microscopy at different pressure (Additional file 1: Fig. S1D).

### Anti-noise ability and bandwidth of the microelectrode in the flexible device

To evaluate the anti-noise ability of microelectrode in a flexible device, the introduced electrical noise testing was performed [60]. The microelectrodes (carbon fiber with nano-copper and the bare carbon fiber) were respectively connected to an amplifying circuit, voltage signal was recorded with time-varying at a sampling frequency of 10 kHz. Anti-noise comparison of the two-type microelectrodes was analyzed by the magnitude and power spectrum of the induced voltage.

To verify whether the bandwidth of the device is enough for capturing respiration and electrical activity of the heart, impedance spectroscopy analysis (Autolab, Switzerland) was used to scan a device from the frequency of 0.1 Hz to 100 kHz. The magnitude of AC voltage was set as 20 mV [74].

### Simultaneous detection of respiration and ECG in the human body

Flexible devices were attached to 17 volunteers, whose ECG and resistance change deduced by respiration and heart electrical activity were both recorded. The signals from volunteers at rest were recorded for 2 min, and continuing to record for 2 min after running. We ethically obligated to perform every test.

### Optimization of nano-copper modification

To obtain the optimal sensitivity for the strain sensor and the electrode in the flexible device, devices were modified for six groups of conditions (5 s, 10 s, 20 s, 40 s, 80 s, and 160 s) at the potential of 12 V. After modification, resistance changes of different devices were compared to find the largest value and its corresponding modification condition was selected for the strain sensor in flexible device. For microelectrode, the optimal modification condition was evaluated by the introduced electrical noise level.

## Conclusion

This paper presents a flexible device for the simultaneous detection of human respiration and cardiac electrical activity. To avoid interference between the two signals, the layout of the electrode and the strain sensor was optimized by FEA simulation analysis. To improve the piezo-resistive sensitivity and bio-electric capturing capability of the device, a feather-shaped nano-copper was modified onto the surface of carbon fiber. The piezo-resistive strain sensor in the device achieved the resolution of 100 Pa in pressure measurement with a sensitivity of 0.053 ± 0.00079 kPa^−1^, The S/N ratio of the voltage improves from 2.2 ± 1.9 to 10.7 ± 1.4. Furthermore, the optimal modification condition and mechanisms of sensing strain and voltage were determined. Finally, the device was applied to measure the signals of 17 volunteers. In future work, we will integrate the signal acquisition and wireless transmission elements in our device.

## Supplementary information

**Additional file 1.** Supplementary figures.

**Additional file 2.** Simultaneous measurement of respiration and electrical activity of a volunteer by using the proposed flexible device.

**Additional file 3.** The preconditioning process of the proposed flexible device by a home-made stretching instrument.

## Data Availability

Materials described in the manuscript, including all relevant raw data, will be freely available to any scientist wishing to use them for non-commercial purposes upon request via e-mail with corresponding author.
