# Supplementary material for: Nano-copper enhanced flexible device for simultaneous measurement of human respiratory and electro-cardiac activities
Source: J Nanobiotechnology. 2020 May 29;18:82. doi: 10.1186/s12951-020-00632-3 (PMC7257177; doi:10.1186/s12951-020-00632-3)

Additional files 3 — supplementary figures

**Nano-copper Enhanced Flexible Device for Simultaneous Measurement of Human Cardio-pulmonary Activities**

Li Wang^1,^*, Feng Zhang^1^, Kechao Lu^1^, Mohammed Abdulaziz^2^, Chao Li^3^, Chongyu Zhang^4^, Jun Chen^1,^*, Yunlun Li^3,^*

^1^School of Mechanical & Automotive Engineering, Qilu University of Technology (Shandong Academy of Sciences), Jinan 250353, China.

^2^Department of Mechanical and Process Engineering, University of Duisburg Essen, Forsthausweg 247057, Germany.

^3^Experimental center, Shandong University of Traditional Chinese Medicine, Jinan, 250355, China.

^4^the Second Affiliated Hospital, Guangzhou Medical University, Guangzhou 510260, China.

* Corresponding author: [chenjun@qlu.edu.cn](mailto:chenjun@qlu.edu.cn), [liwang@qlu.edu.cn](mailto:liwang@qlu.edu.cn), yunlun.lee@hotmail.com; Tel.: +86-0531-8963-1702; Fax: +86-0531-8963-1702.

Fig.S1 (A) The flexible device with nano-copper and without nano-copper was preconditioned for 6000 s. The bottom plots was zoomed for demonstrating the resistance changes of the device without nano-copper (bottom-left) and the device without nano-copper (bottom-right). (B) The schematic for showing the setup of calibrating resistance change and pressure. (D) The flexible device was bulged under different pressures. (E) The time domain graph of the introduced background noise.


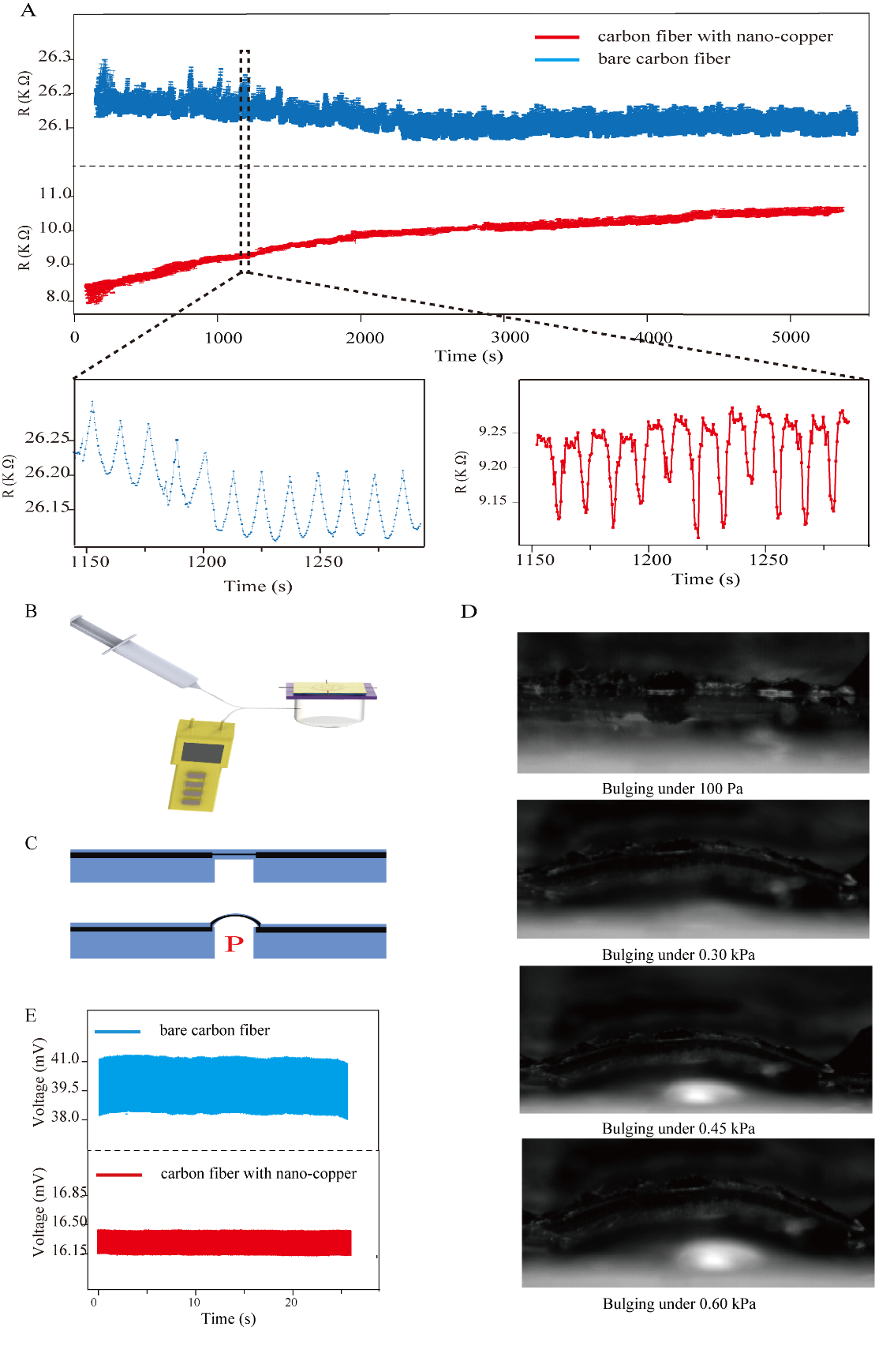


Fig.S2 The detailed fabrication process of the flexible device.


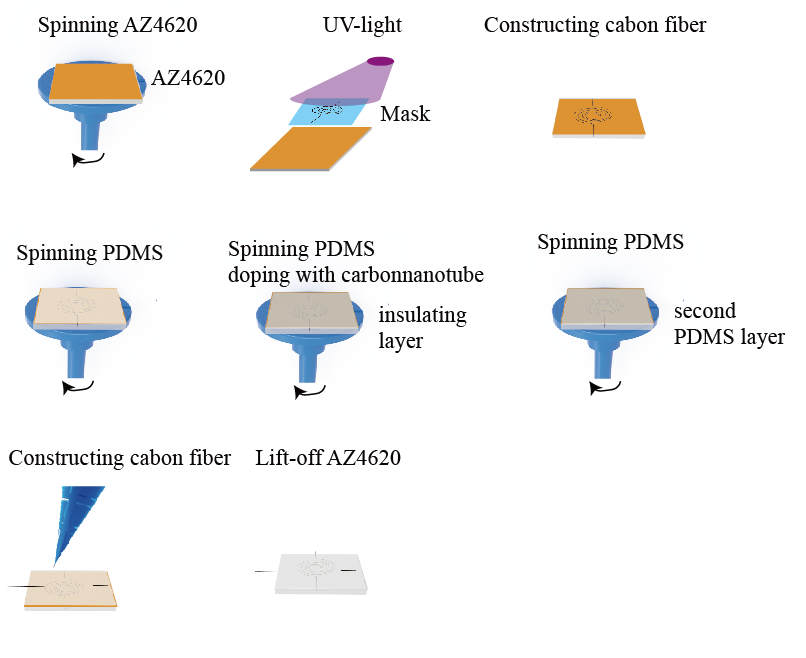


Fig. S3（A）AFM was used to test young’s modulus of PDMS and PDMS inserted with carbon fiber. Both young’s modulus were 467.5 ± 10.27 kPa (B) A home-made streching instrument for preconditioning flexible device. (C) (D) The calibration ships between the spinning speed and the thickness of AZ4620 and PDMS.


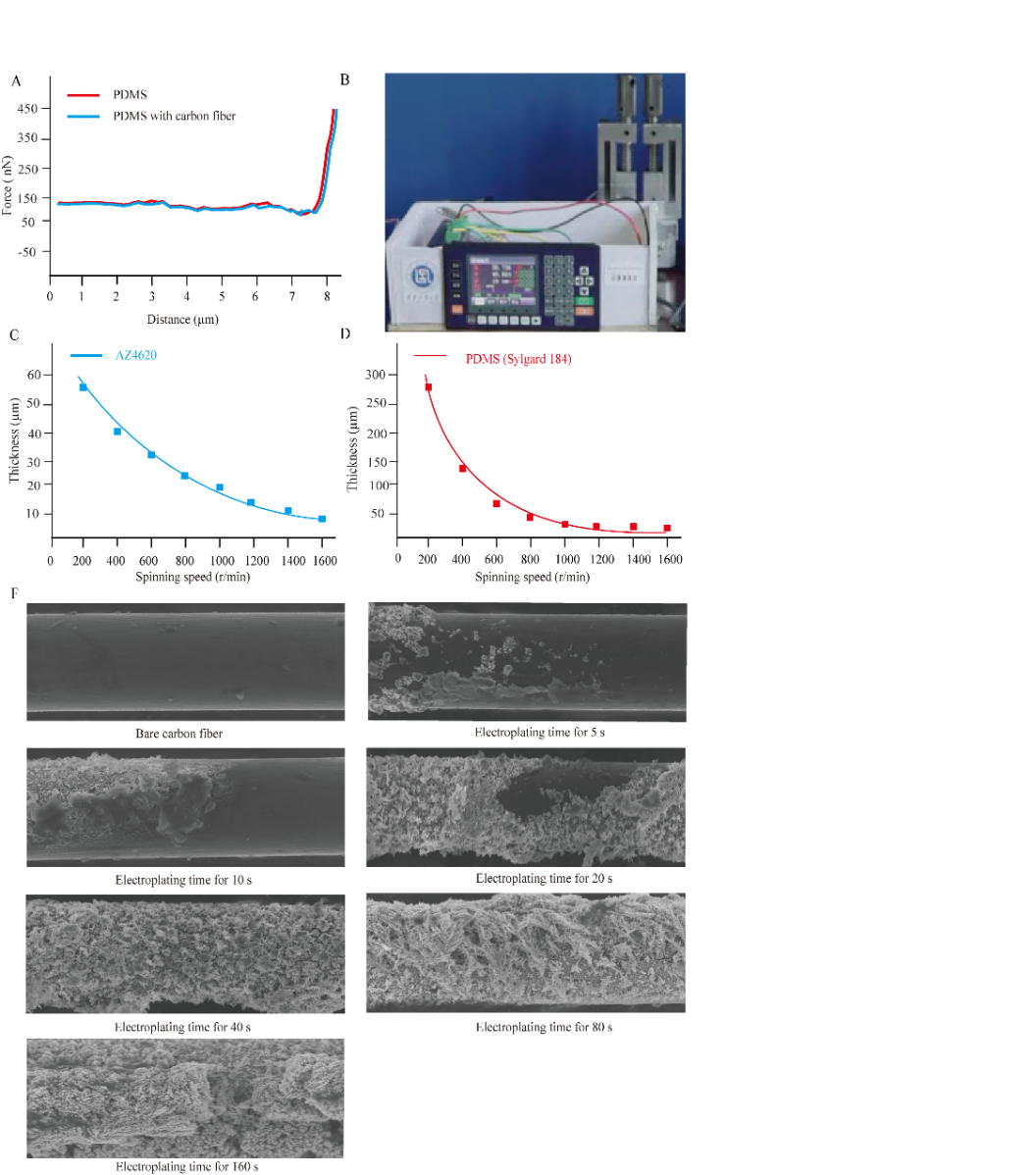

Supplement: Supplementary file 1 — Additional file 1. Supplementary figures. [file 12951_2020_632_MOESM1_ESM.docx]
